# Maternal Anthropometric and Obstetric Risk Factors for Small-for-Gestational-Age Births in Anuradhapura District, Sri Lanka: A Retrospective Case-Control Study

**DOI:** 10.5334/aogh.5330

**Published:** 2026-06-15

**Authors:** G. N. Duminda Guruge, Johanna Enberg, Oshini Sri Jayasinghe, Kalpani Abhayasinghe, Hakan Lilja

**Affiliations:** 1 Department of Health Promotion, Faculty of Applied Sciences, Rajarata University of Sri Lanka, Mihintale, Sri Lanka; 2 Department of Gynecology and Obstetrics, Sahlgrenska Academy, University of Gothenburg, Gothenburg, Sweden; 3 Department of Nursing and Midwifery, Faculty of Allied Health Sciences, General Sir John Kotelawala Defense University, Ratmalana, Sri Lanka

**Keywords:** small for gestational age, maternal nutrition, body mass index, low birth weight, case-control study, antenatal care, Sri Lanka, low- and middle-income countries

## Abstract

*Background:* Small-for-gestational-age (SGA), defined as birth weight below the 10th percentile for gestational age, is a major cause of neonatal morbidity and mortality, disproportionately affecting low- and middle-income countries (LMICs). The Anuradhapura district of Sri Lanka carries the highest low birth weight (LBW) prevalence nationally, yet no case-control study has systematically characterized independent maternal risk factors for SGA in this population.

*Objectives:* To identify maternal anthropometric, obstetric, and antenatal risk factors independently associated with SGA births in Anuradhapura district, Sri Lanka.

*Methods:* A retrospective, matched case-control study was conducted using Public Health Midwife (PHM) pregnancy records from 2014 to 2017 across two Medical Officer of Health (MOH) areas. Cases (*n* = 136) were SGA infants (birth weight < 10th percentile for gestational age) individually matched 1:2 to average-for-gestational-age (AGA) controls (*n* = 272) by gestational age category. Multivariable binary logistic regression with two separate models identified independent predictors.

*Findings:* Five independent maternal risk factors were identified. Prepregnancy weight <50 kg (adjusted OR 2.18; 95% CI, 1.28–3.69; *P* = 0.004), prepregnancy BMI <18.5 kg/m² (OR 2.24; 95% CI, 1.27–3.94; *P* = 0.005), prepregnancy BMI ≥25 kg/m² (OR 1.95; 95% CI, 1.04–3.64; *P* = 0.036), maternal height ≤150 cm (OR 1.98; 95% CI, 1.14–3.45; *P* = 0.015), and history of a prior LBW infant (OR 3.87; 95% CI, 1.98–7.57; *P* < 0.001) were independently associated with SGA. Borderline associations for consanguinity, anemia, and absence of folic acid supplementation did not persist in adjusted models.

*Conclusions:* Both undernutrition and overweight independently elevate SGA risk, reflecting a double burden of malnutrition in this LMIC setting. A prior LBW delivery is the single strongest predictor, underscoring intergenerational risk pathways. Preconception nutritional screening and targeted antenatal interventions are essential to reduce the SGA burden in Anuradhapura and comparable settings.

## Introduction

Low birth weight (LBW), defined as birth weight <2500 g, is a leading driver of neonatal mortality globally, accounting for an estimated 60%–70% of all neonatal deaths [1]. Beyond mortality, LBW is strongly associated with impaired neurodevelopmental outcomes, childhood morbidity, stunting, and adult-onset noncommunicable diseases [2, 3]. An estimated 15.5% of all births worldwide are LBW, representing >20 million births annually, with >95% occurring in low- and middle-income countries (LMICs) [1, 4]. Preterm birth and small-for-gestational-age (SGA) are the two principal etiological pathways to LBW [4, 5].

SGA is defined as birth weight below the 10th percentile or >2 standard deviations below the population mean, for a given gestational age [6]. In LMICs, the burden of SGA disproportionately exceeds that of preterm birth, yet context-specific evidence to guide prevention remains limited [5, 7].

Established maternal risk factors for SGA include undernutrition and low prepregnancy weight [8], iron-deficiency anemia [9], chronic hypertension, and hypertensive disorders of pregnancy [10, 11]. Short maternal stature is recognized as a biological marker of intergenerational nutritional deprivation [12]. Emerging evidence implicates maternal overweight in SGA risk, potentially mediated through vascular and metabolic complications [13].

In Sri Lanka, substantial gains in maternal mortality have been achieved over recent decades; however, maternal and child malnutrition persist, particularly in the North Central, Northern, and Eastern provinces [12, 14, 15]. The Anuradhapura district recorded an LBW prevalence of ~17% in 2011 (1966 of 11,560 live births) [16] and consistently reports the highest LBW rates nationally [14]. Despite this burden, no matched case-control study has previously characterized independent maternal risk factors for SGA in this population.

This study aimed to identify maternal anthropometric, obstetric, and antenatal risk factors independently associated with SGA births in Anuradhapura district, Sri Lanka, to inform targeted, evidence-based antenatal care strategies.

## Methods

### Study design and setting

A retrospective case-control study was conducted using pregnancy records maintained by Public Health Midwives (PHMs) in 13 areas across two Medical Officer of Health (MOH) areas in Anuradhapura district, Sri Lanka. PHMs are community-based maternal and child health workers each responsible for populations of 3000–5000 persons. Data were extracted from PHM records spanning January 2014 to December 2017. This study is reported in accordance with the Strengthening the Reporting of Observational Studies in Epidemiology (STROBE) statement [17].

### Case and control definitions

Cases were neonates with birth weight below the 10th percentile for gestational age (SGA). Controls were neonates with birth weight between the 10th and 90th percentile (AGA). All SGA infants registered with participating PHMs during the study period were eligible as cases. Neonates from multiple pregnancies, those born after 43 completed weeks, large-for-gestational-age infants, and stillbirths were excluded. Two AGA controls were individually matched to each case (1:2 ratio), yielding 136 cases and 272 controls (*N* = 408). Matching was performed by gestational age category: extremely preterm (<30 + 0 weeks), preterm (30 + 0–36 + 6 weeks), term (37 + 0–41 + 6 weeks), and postterm (>= 42 + 0 weeks). Within each category, controls born in the same gestational week as the case were preferentially selected, and all controls were restricted to those born within one year of the corresponding case.

### SGA ascertainment

Gestational age was calculated from the first day of the last menstrual period, with corroborating ultrasound measurements used when available (<20 weeks). Birth weight percentiles were determined using a computer-based reference chart based on the Hadlock fetal weight equation [18, 19] calibrated to a Sri Lankan population mean birth weight of 3140 g at 40 weeks of gestation [20].

### Data extraction

A structured data extraction form was developed based on a systematic review of global literature on SGA determinants. Variables captured included maternal sociodemographic characteristics (age, education, occupation, parity, and marital status), anthropometric measurements (prepregnancy weight, height, body mass index (BMI), and gestational weight gain), obstetric history (previous LBW delivery, miscarriage, subfertility, cesarean section, and consanguinity), and antenatal factors (folic acid supplementation, anemia, gestational hypertension, preeclampsia, antepartum hemorrhage, and mode of delivery). All records were assigned anonymized identification numbers.

### Statistical analysis

Data were entered and analyzed using IBM SPSS Statistics version 24. Categorical variables were examined using Pearson chi-square test or Fisher exact test when expected cell counts were <5. Variables with *P* < 0.10 in unadjusted analyses were advanced to multivariable logistic regression. Pairwise Spearman rank correlations among candidate variables all yielded coefficients <0.20, confirming mutual independence.

Given collinearity between prepregnancy weight, height, and BMI, two separate multivariable models were constructed: Model 1 included prepregnancy weight and height; Model 2 included prepregnancy BMI. Both models adjusted a priori for infant sex, maternal age, parity, and educational level. Adjusted ORs with 95% CIs are reported. Model fit was evaluated using the Hosmer–Lemeshow goodness-of-fit test. Statistical significance was set at *P* < 0.05 (two-tailed).

Per the AMA Manual of Style, all *P* values are reported without a leading zero, with results reported as statistically significant when *P* < 0.05 and as statistically nonsignificant when *P* >= 0.05.

### Ethical considerations

Ethical approval was granted by the Ethics Review Committee of the Faculty of Applied Sciences, Rajarata University of Sri Lanka (approval date: January 30, 2017; permit number: ERC/FAS/2017/01). Individual informed consent was waived as the study involved secondary analysis of de-identified, routinely collected PHM records, in accordance with applicable national ethical guidelines. All procedures complied with the Declaration of Helsinki (2013 revision).

## Results

### Study population

A total of 408 PHM records were analyzed (136 SGA cases and 272 AGA controls). Of all infants, 51.7% (*n* = 215) were male and 46.4% (*n* = 193) were female (1.9% gender data missing). Participants were drawn from MOH Area A (53.7%; *n* = 219) and MOH Area B (46.3%; *n* = 189). All mothers were married. The SGA prevalence in the study population was 5.4%. Of SGA infants, 57.4% (*n* = 78) were below the 10th percentile, 14.7% (n = 20) below the 5th percentile, and 27.9% (*n* = 38) below the 3rd percentile. Approximately 11% of SGA infants were preterm; no extremely preterm or postterm infants were identified. Maternal and neonatal characteristics are presented in Table 1.

**Table 1:** Maternal and neonatal clinical characteristics of the study sample (*N* = 408).

CHARACTERISTIC	CONTROLS (AGA), *N* = 272			CASES (SGA), *N* = 136			TOTAL, *N* = 408		
	MEAN (SD)	MIN	MAX	MEAN (SD)	MIN	MAX	MEAN (SD)	MIN	MAX
Birth weight (g)	2806 (319)	1446	3600	2257 (329)	700	2760	2623 (413)	700	3600
Gestational age (weeks)	38 (2)	31	41	39 (2)	30	41	38 (2)	30	41
Maternal age (years)	28 (6)	16	41	27 (5)	17	44	28 (6)	16	44
Prepregnancy weight (kg)*	52.7 (10.5)	34.0	85.0	49.1 (11.5)	30.5	83.6	51.6 (10.9)	30.5	85.0
Maternal height (cm)**	155 (6)	142	172	152 (6)	139	180	154 (6)	139	180
Prepregnancy BMI (kg/m^2^)	21.9 (4.2)	13.6	37.3	21.1 (4.8)	12.7	37.2	21.7 (4.4)	12.7	37.3
Gestational weight gain (kg)	9.7 (4.1)	1.0	23.6	9.5 (4.5)	1.7	22.0	9.7 (4.2)	1.0	23.6

*Abbreviations*: AGA, average-for-gestational-age; BMI, body mass index; SD, standard deviation; SGA, small-for-gestational-age. *48 records (11.8%) missing prepregnancy weight. **12 records (2.9%) missing height data.

### Unadjusted associations

Chi-square analysis identified significant unadjusted associations between SGA and pre-pregnancy weight (*P* < 0.001), maternal height (*P* = 0.007), and prepregnancy BMI (*P* = 0.003). A history of a previous LBW infant was also significantly associated with SGA (*P* < 0.001). Mode of delivery differed significantly between groups (*P* = 0.012), with cesarean section more frequent among SGA cases (41.9%) than AGA controls (29.4%). Anemia (*P* = 0.067) and absence of folic acid supplementation (*P* = 0.062) showed borderline associations. No significant associations were found for parity, previous miscarriage, gestational weight gain, gestational hypertension, or antepartum hemorrhage (all *P* > 0.10). Complete results are presented in Table 2.

**Table 2:** Unadjusted univariate analysis of maternal and antenatal factors associated with SGA (*N* = 408).

VARIABLE	TOTAL (*N* = 408) *N* (%)	SGA CASES (*N* = 136) *N* (%)	AGA CONTROLS (*N* = 272) *N* (%)	*P* VALUE
**Maternal anthropometric factors**				
Prepregnancy weight (kg)				<0.001^a^
<50 kg	165 (45.8)	68 (59.1)	97 (39.6)	
>= 50 kg (reference)	195 (54.2)	47 (40.9)	148 (60.4)	
Maternal height (cm)				0.007^a^
<= 150 cm	118 (29.2)	52 (38.8)	66 (24.4)	
151–160 cm	229 (56.7)	69 (51.5)	160 (59.3)	
>160 cm	57 (14.1)	13 (9.7)	44 (16.3)	
Prepregnancy BMI (kg/m^2^)				0.003^a^
<18.5 (underweight)	93 (26.0)	41 (36.0)	52 (21.3)	
18.5–24.9 (normal; reference)	185 (51.7)	45 (39.5)	140 (57.4)	
>= 25 (overweight/obese)	80 (22.3)	28 (24.6)	52 (21.3)	
**Obstetric history**				
Parity (primiparous)	170 (40.9)	63 (46.3)	107 (39.3)	0.177
History of subfertility	13 (3.1)	7 (5.1)	6 (2.2)	0.136
Previous LBW infant	74 (17.8)	40 (29.4)	34 (12.5)	<0.001^a^
Previous miscarriage	64 (15.4)	21 (15.4)	43 (15.8)	0.923
Previous cesarean section	52 (12.5)	14 (10.3)	38 (14.0)	0.294
Consanguinity	13 (3.1)	7 (5.1)	6 (2.2)	0.136
**Antenatal and delivery factors**				
Anemia in pregnancy	111 (26.7)	45 (33.8)	66 (25.1)	0.067
Folic acid supplementation (yes)	274 (65.9)	83 (61.0)	191 (70.2)	0.062
Gestational weight gain				0.471
Below recommended	170 (47.8)	58 (51.3)	112 (46.1)	
Within recommended	133 (37.4)	37 (32.7)	96 (39.5)	
Above recommended	53 (14.9)	18 (15.9)	35 (14.4)	
Antepartum hemorrhage	9 (2.2)	4 (2.9)	5 (1.8)	0.489^b^
Gestational hypertension	11 (2.6)	4 (3.0)	7 (2.6)	1.000^b^
Mode of delivery (cesarean section)	137 (33.6)	57 (41.9)	80 (29.4)	0.012^a^

*Abbreviations*: AGA, average-for-gestational-age; LBW, low birth weight; LSCS, lower segment cesarean section; SGA, small-for-gestational-age. ^a^*P* < 0.05 (statistically significant). ^b^Fisher exact test applied.

### Multivariable logistic regression

Both multivariable models confirmed independent associations between maternal anthropometric factors and SGA (Table 3). In Model 1, prepregnancy weight <50 kg (adjusted OR 2.18; 95% CI, 1.28–3.69; *P* = 0.004) and maternal height <= 150 cm (adjusted OR 1.98; 95% CI, 1.14–3.45; *P* = 0.015) were significant independent predictors. In Model 2, both underweight BMI <18.5 kg/m^2^ (adjusted OR 2.24; 95% CI, 1.27–3.94; *P* = 0.005) and overweight BMI >= 25 kg/m^2^ (adjusted OR 1.95; 95% CI, 1.04–3.64; *P* = 0.036) were significantly associated with SGA. Across both models, a prior LBW delivery was the strongest predictor (adjusted OR 3.87; 95% CI, 1.98–7.57 in Model 1 and 2.01–7.47 in Model 2; both *P* < 0.001). Consanguinity, folic acid supplementation, and anemia did not reach significance in either adjusted model.

**Table 3:** Multivariable binary logistic regression: adjusted odds ratios for maternal risk factors of SGA (*N* = 408).

MODEL	VARIABLE	ADJUSTED OR	95% CI LOWER	95% CI UPPER	*P* VALUE
**Model 1**	Prepregnancy weight <50 kg (ref: >= 50 kg)	2.18	1.28	3.69	0.004^a^
	Height < = 150 cm (ref: 151-160 cm)	1.98	1.14	3.45	0.015^a^
	Height > 160 cm (ref: 151–160 cm)	0.82	0.37	1.82	0.615
	Previous LBW infant	3.87	1.98	7.57	<0.001^a^
	Consanguinity	1.95	0.69	5.58	0.210
	Folic acid supplementation	1.44	0.84	2.49	0.186
	Anemia in pregnancy	1.37	0.77	2.41	0.285
**Model 2**	BMI < 18.5 kg/m^2^ (ref: 18.5–24.9 kg/m^2^)	2.24	1.27	3.94	0.005^a^
	BMI >= 25 kg/m^2^ (ref: 18.5–24.9 kg/m^2^)	1.95	1.04	3.64	0.036^a^
	Previous LBW infant	3.87	2.01	7.47	<0.001^a^
	Consanguinity	1.61	0.59	4.44	0.354
	Folic acid supplementation	1.33	0.78	2.28	0.295
	Anemia in pregnancy	1.32	0.76	2.32	0.325

*Abbreviations*: BMI, body mass index; CI, confidence interval; LBW, low birth weight; OR, odds ratio; SGA, small-for-gestational-age. Both models adjusted a priori for infant sex, maternal age, parity, and educational level. Reference categories: weight >= 50 kg; height 151–160 cm; BMI 18.5–24.9 kg/m^2^. ^a^Statistically significant (*P* < 0.05).

## Discussion

This retrospective case-control study identified five independent maternal risk factors for SGA births in Anuradhapura district, Sri Lanka: low prepregnancy weight, short maternal stature, underweight BMI, overweight BMI, and a prior LBW delivery. These findings are broadly consistent with global evidence linking maternal body composition to fetal growth restriction [21–24] and align with earlier Sri Lankan research documenting maternal undernutrition and pregnancy-induced hypertension as determinants of adverse birth outcomes [8].

Associations between maternal undernutrition (low prepregnancy weight, short stature, and underweight BMI) and SGA are mechanistically well-established: inadequate prepregnancy energy and nutrient reserves restrict placental development and fetal substrate delivery [8, 21, 22]. Short maternal stature is a recognized biological marker of cumulative intergenerational nutritional deprivation and childhood poverty, both prevalent in the Anuradhapura district [12, 25].

The finding that overweight BMI (>= 25 kg/m^2^) also elevated SGA risk warrants careful interpretation. While some studies report null or protective effects of maternal overweight on SGA [23, 24], others document increased risk mediated through hypertensive disorders, gestational diabetes, or placental vascular insufficiency [13, 25]. Our study lacked statistical power to examine these mediators directly, but the observed association is consistent with the double burden of malnutrition increasingly documented in South Asian LMICs, where overweight coexists with micronutrient deficiency and compromised metabolic health [26].

The strongest predictor in both models was a prior LBW delivery (adjusted OR approximately 3.87). This reflects both genetic determinism and recurrent socioeconomic and nutritional disadvantage driving intergenerational SGA risk [24, 27]. It reinforces a biological–socioeconomic feedback loop in which maternal growth restriction perpetuates adverse birth outcomes across generations [12, 28].

Consanguinity, anemia, and absence of folic acid supplementation showed borderline univariate trends that did not persist in adjusted models [29–32], likely reflecting insufficient power to detect modest effects. The higher cesarean section rate among SGA cases (41.9% vs 29.4%; *P* = 0.012) reflects obstetric decision-making in response to identified fetal growth restriction rather than a causal antecedent of SGA.

### Strengths and limitations

Strengths include the matched case-control design with gestational age category matching, use of a Sri Lankan population-specific birth weight reference, inclusion of two demographically diverse MOH areas, and the relatively large community-based sample. Retrospective use of PHM records provided real-world programmatic data.

Limitations include missing data (11.8% missing prepregnancy weight; 2.9% missing height), potential gestational age misclassification from last menstrual period-based dating, and inability to capture dietary assessment, socioeconomic status, maternal workload, or micronutrient status. Residual confounding cannot be excluded. The retrospective design precludes causal inference, and restriction to one district limits generalizability.

### Policy implications

These findings support an integrated, life-course approach to maternal health. Preconception and early antenatal screening should routinely assess weight, height, and BMI, with standardized referral for both underweight and overweight women to nutritional counseling and supplementation. Women with a prior LBW delivery are a high-priority group for enhanced antenatal surveillance. Structural drivers of chronic maternal undernutrition in this predominantly rural district require intersectoral policy action addressing food security, female education, and agricultural workloads.

## Conclusions

Maternal undernutrition, short stature, overweight BMI, and a prior LBW delivery are significant independent predictors of SGA in Anuradhapura district, Sri Lanka. The co-occurrence of undernutrition and overweight as independent risk factors reflects the double burden of malnutrition in this LMIC setting. Intergenerational risk pathways, particularly a prior LBW delivery, represent the most actionable target within existing antenatal care infrastructure. Prospective studies with larger samples and comprehensive nutritional and socioeconomic data are needed to build the causal evidence base for scalable interventions.

## Data Availability

The datasets underlying the results of this study are derived from de-identified, routinely collected Public Health Midwife pregnancy records in Anuradhapura district, Sri Lanka, and are not publicly available owing to institutional data governance requirements. Anonymized data may be made available from the corresponding author upon reasonable request, subject to the approval of the relevant institutional authority. Requests should be directed to: dumindaguruge@gmail.com.
